# Symptom Clusters by Edmonton Symptom Assessment System in Radiotherapy and Palliative Care Clinic

**DOI:** 10.3390/medicina62071216

**Published:** 2026-06-23

**Authors:** Lucia Angelini, Andrea Roncadori, Luca Tontini, Martina Pieri, Paola Cravero, Linda Petrini, Margherita Currà, Vanessa Valenti, William Balzi, Valentina Danesi, Ilaria Massa, Marco Cesare Maltoni, Romina Rossi

**Affiliations:** 1 Palliative Care Unit, IRCCS Istituto Romagnolo per lo Studio dei Tumori (IRST) “Dino Amadori”, 47014 Meldola, Italy; lucia.angelini@irst.emr.it (L.A.); paola.cravero@irst.emr.it (P.C.); linda.petrini@irst.emr.it (L.P.); margherita.curra@irst.emr.it (M.C.); vanessa.valenti@irst.emr.it (V.V.); 2 Outcome Research, IRCCS Istituto Romagnolo per lo Studio dei Tumori (IRST) “Dino Amadori”, 47014 Meldola, Italy; william.balzi@irst.emr.it (W.B.); valentina.danesi@irst.emr.it (V.D.); ilaria.massa@irst.emr.it (I.M.); 3 Radiotherapy Unit, IRCCS Istituto Romagnolo per lo Studio dei Tumori (IRST) “Dino Amadori”, 47014 Meldola, Italy; luca.tontini@irst.emr.it (L.T.); martina.pieri@irst.emr.it (M.P.); 4 Department of Medical and Surgical Sciences (DIMEC), University of Bologna, 40126 Bologna, Italy; marcocesare.maltoni@unibo.it (M.C.M.); romina.rossi10@unibo.it (R.R.)

**Keywords:** symptom clusters, palliative radiotherapy, ESAS, advanced cancer, decision-making, multidisciplinary care

## Abstract

*Background and Objectives*: Effective palliative care relies on accurate identification and management of symptoms, especially in patients referred for palliative radiotherapy (PRT). This study aimed to identify symptom clusters (SCs)—defined as ≥2 interrelated symptoms—in patients evaluated at a multidisciplinary Radiotherapy and Palliative Care (RaP) outpatient clinic, using the Edmonton Symptom Assessment System (ESAS). *Materials and Methods*: We retrospectively analyzed data from patients referred to the RaP clinic between February 2017 and April 2020. Demographic and clinical characteristics, including ESAS scores at first visit, were collected. SCs were identified with principal component analysis (PCA) and unsupervised k-means clustering (KMC), determining the number of SCs based on the maximum gap statistic and interpretability. Associations with ECOG performance status (PS), primary tumor and metastases site, and PRT administration were analyzed. Exploratory survival analyses were performed. *Results*: Among 215 patients (median age = 71 years; 53% male), the mean total ESAS score was 24.03 (SD = 15.28). PCA identified four SCs: SC_PCA_1 (tiredness, drowsiness, dyspnea, malaise), SC_PCA_2 (depression, anxiety), SC_PCA_3 (nausea, loss of appetite) and SC_PCA_4 (pain). KMC revealed three SCs: SC_KMC_1 (pain, tiredness, drowsiness, malaise), SC_KMC_2 (nausea, loss of appetite, dyspnea), and SC_KMC_3 (depression, anxiety). Worse ECOG PS correlated with physical SCs (*p* < 0.05). Psychological SCs were associated with lower likelihood of receiving PRT (OR_PCA2_ = 0.26, CI: 0.07–0.80, OR_kmc3_ = 0.19, CI: 0.02–0.85, *p* < 0.05), but when associated with pain/systemic clusters correlated with greater PRT use. A trend toward shorter survival was seen in SC_KMC_2. *Conclusions*: SC analysis could improve personalized symptom management and clinical decision-making in the PRT setting.

## 1. Introduction

Palliative radiotherapy (PRT) plays a pivotal role in the management of advanced cancer patients, offering relief from symptoms such as pain, bleeding, and neurological deficits caused by tumor progression [1,2,3]. These patients frequently present with a high symptom burden, arising both from the underlying malignancy and the adverse effects of systemic therapies [4,5]. Beyond the physical domain, psychological distress is highly prevalent, often resulting from a complex interplay of poorly controlled symptoms, existential concerns, and the emotional impact of an incurable illness [6,7,8]. Clinical decision-making regarding the administration of PRT is inherently complex, involving appropriateness, timing, and dose-fractionation considerations. A comprehensive, patient-centered approach, that integrates radiation oncology with palliative care, promotes holistic assessment and shared decision-making [9,10]. Internationally, structured models such as the Rapid Response Radiotherapy Program in Toronto have demonstrated that integrating palliative and supportive care within radiation oncology is feasible and enhances the timeliness, appropriateness, and quality of PRT delivery [11]. In Italy, despite recommendations from the Italian Association of Radiation Oncology (AIRO) emphasizing patient selection and the avoidance of non-beneficial treatments [12], national integrated models remain limited. A survey conducted by the Italian Association of Medical Oncology (AIOM) highlighted unmet symptom needs and strong interest among oncologists in earlier collaboration with palliative care; however, robust data on symptom burden and the impact of integrated models in the Italian context are still scarce [13]. To address this need, our institution established the Radiotherapy and Palliative Care (RaP) outpatient clinic, a multidisciplinary service in which radiation oncologists and palliative care specialists collaborate in the evaluation and management of patients referred for PRT. Preliminary experience from this clinic has demonstrated improvements in care quality, including more appropriate patient selection for radiotherapy and timely access to palliative care services [14].

To further enhance individualized care, it is crucial to consider symptoms as interrelated phenomena that may reflect underlying syndromic patterns. The concept of symptom clusters (SC)—defined as groups of two or more concurrent and interrelated symptoms with potential shared pathophysiology—has gained prominence as a framework for understanding the multidimensional symptom burden in advanced cancer [15,16,17,18,19,20,21].

Identifying SCs may shed light on the shared mechanisms behind symptom co-occurrence and may support more effective symptom management strategies. Among the most widely used tools for symptom assessment in oncology is the Edmonton Symptom Assessment System (ESAS), a validated instrument that evaluates nine symptoms (pain, tiredness, drowsiness, nausea, loss of appetite, dyspnea, depression, anxiety and well-being), plus an optional tenth symptom that can be reported by the patient, using a numerical rating scale (NRS) [22]. This secondary analysis of the RaP study by Rossi et al. [14] aims to retrospectively identify SCs through ESAS in patients referred for PRT. We hypothesized that distinct SCs could be identified in this population and that these clusters may differ in terms of performance status, treatment allocation, and clinical outcomes.

## 2. Materials and Methods

This study represents a secondary, post hoc retrospective cohort analysis of the RaP clinic database. The RaP clinic is a joint outpatient service where radiation oncologists and palliative care physicians evaluate patients together to assess indications for PRT and supportive care needs. Before each consultation, a specifically trained nurse administers the ESAS questionnaire, assisting patients in understanding and completing the assessment when necessary. The nurse also performs an initial symptom-focused triage, providing a preliminary evaluation of symptom burden that informs the subsequent multidisciplinary discussion. Patients with metastatic disease from any primary tumor are referred to the clinic, where both specialists perform a simultaneous clinical assessment. Treatment-related needs and patient goals of care are discussed in a multidisciplinary setting, and a shared decision is made regarding optimization of supportive care (including adjustment of analgesic and symptom control therapies), indication for PRT, or a combination of both approaches.

All patients assessed in the RaP clinic between February 2017 and April 2020 were included. All patients were asked to complete the Italian version of the Edmonton Symptom Assessment System, validated by Moro et al. in 2006 [23], in the presence of a specialist nurse. No additional inclusion or exclusion criteria were applied, and all included patients had complete ESAS data, with no missing questionnaires. Patients of all ages and all primary tumor types were eligible for inclusion. For each patient, sociodemographic variables (sex and age at the first RaP visit) and clinical characteristics were collected. Clinical data included ECOG PS, as independently assessed by both radiation oncologist and palliative care physician. Disease-related variables included primary tumor site, presence of locally advanced disease, and sites of metastases. Symptom burden was assessed using ESAS. The ‘Other symptom’ field was available in the version used; however, due to the heterogeneity of responses, this item was not included in the statistical analyses.

The original study was approved by the IRCCS-IRST and Wide Area Romagna Ethics Committee (CEIIAV), approval Code n.1517 on 17 December 2015.

### Statistical Analysis

Demographic and baseline clinical data was summarized by means of absolute frequencies and relative percentages. To describe each ESAS item, mean, standard deviation (SD) and the proportion of patients presenting a score greater or equal to four were reported. Furthermore, SCs were identified using both principal component analysis (PCA) and unsupervised k-means clustering (KMC). In practice, to explore potential patterns of association (i.e., linear combinations) between ESAS items, a PCA with varimax rotation was performed. Indeed, the PCA transforms several observed variables into a reduced number of variables called principal components. Pragmatically, prior to the analysis, all variables were standardized to ensure comparability and minimize scale-related bias. Sampling adequacy was assessed using the Kaiser–Meyer–Olkin (KMO) measure; additionally, the suitability of the correlation matrix for factorization was evaluated using Bartlett’s test of sphericity. Both tests confirmed the appropriateness of the dataset for principal component analysis. Essentially, the varimax rotation was used to maximize the variance of a column of the factor pattern matrix. Significant principal components were selected with an eigenvalue higher than 0.8, a threshold chosen to avoid excluding clinically meaningful but moderately expressed symptom dimensions. In addition, each retained component explained at least 10% of the variance. The highest factor loading score was used for assigning the ESAS items to an independent factor. The set of items assigned to the same independent factor collectively compose a cluster. For sensitivity purposes, a PCA using oblique (Promax) rotation was also conducted. The results were highly consistent across rotation methods, supporting the robustness of the identified symptom clusters. Robust relationships and correlations among symptoms were displayed with the biplot representation. Subsequently, unsupervised KMC was applied at the level of ESAS symptom variables (i.e., variable space rather than patient-level clustering) to identify natural groupings in ESAS symptom space. The optimal number of SCs was determined using a combination of statistical methods, namely the Elbow method (within-cluster sum of squares), the Silhouette method and the Gap Statistic. The final number of SCs was selected based on the maximum gap statistic and interpretability, resulting in a three-cluster solution. To further interpret SC structures, between-cluster variance and R2 statistics were computed for each variable. R2 for each variable was calculated as the ratio of between-cluster sum of squares to total sum of squares. Additionally, a separation metric was derived to compare each variable’s fit within its assigned cluster versus the best alternative cluster. Consequently, each ESAS symptom was assigned to the cluster with the highest R^2^ value (i.e., maximum between-cluster separation).

To investigate the association between SCs and the likelihood of receiving PRT, logistic regression models were developed. The binary outcome variable was defined as receipt of PRT (1 = yes; 0 = no). Predictor variables included membership in SCs as derived from principal component analysis (SC_PCA_1–SC_PCA_4) and k-means cluster analysis (SC_KMC_1–SC_KMC_3). Specifically, patients were assigned to a symptom cluster when at least half of the symptoms composing that cluster had clinically relevant intensity, defined as an ESAS score ≥ 4 on the 0–10 numerical rating scale (NRS).

Univariate logistic regressions were first performed to identify potential predictors of PRT, with variables showing *p* < 0.20 considered for inclusion in the multivariable analysis. Multivariable models were then estimated to assess whether SCs were independently associated with PRT after adjusting for relevant clinical covariates. To address small-sample bias and reduce the risk of overfitting, Firth penalized logistic regression was employed. Coherently, final model coefficients were exponentiated and reported as odds ratios (OR) with 95% confidence intervals (CI), alongside profile penalized Likelihood Ratio Test (LRT) *p*-values and type III *p*-values for multi-level predictors (e.g., primary tumor site). Interestingly, when examining RaP clinicians’ propensity to prescribe PRT, selected interaction terms between symptom clusters were also included in the model. Specifically, given the limited sample size and the risk of overparameterization, interactions considered clinically meaningful (and supported by the exploratory analyses) were assessed in the multivariable models. Intuitively, this suggests that a mixture of co-occurring symptoms may influence therapeutic decisions. Moreover, to assess associations between SCs and clinical characteristics (e.g., ECOG status, primary tumor site, metastatic locations), chi-square tests of independence were conducted. Furthermore, for the development of the final models with the aim of identifying a parsimonious model while limiting overfitting, regressors selection was performed using an Akaike Information Criterion (AIC)-based stepwise procedure. Finally, Kaplan–Meier survival curves were generated to compare overall survival (OS) across PCA and KMC groups. OS was defined as the time from the first RaP consultation to death or last follow-up, expressed in months. Survival curves were drawn separately for each SC and grouped by patients belonging to each cluster. Moreover, to further examine prognostic factors associated with OS, Cox proportional hazards regression models with Firth-type penalization were developed. Baseline variables with a *p*-value < 0.20 in univariate models were considered for the inclusion in the multivariable models. Interaction terms between SCs and key clinical covariates were also tested. In detail, the same modeling approach was applied separately for symptom clusters derived from PCA and k-means. Final model results are reported as hazard ratios (HR) with their 95%CIs and profile penalized *p*-values. Moreover, type III *p*-values were also reported for multi-level predictors.

All analyses were performed using R Statistical Software (version 4.4.2). The ‘psych’, ‘factoextra’ and ‘cluster’ libraries were used for performing the PCA and k-means cluster analysis. Finally, ‘ggplot2’ package was used for drawing the figures and graphs.

## 3. Results

In the 215 patients included in the present analysis, the median age was 71 years (range: 37–90), and 122 (53%) were male. ECOG PS was 0–1 in 102 patients (47%) and ≥2 in 113 patients (53%). As ECOG PS was fully concordant between the two clinicians in the RaP clinic, a single score per patient was considered. Lung cancer was the most common primary tumor (n = 68, 31%), while bone was the most frequent metastatic site (n = 106, 49%). Detailed demographic and clinical characteristics are summarized in Table 1.

The mean total ESAS score was 24.03 (SD = 15.28; range = 0–80). Tiredness and pain had the highest mean NRS values (M = 4.91, SD = 3.04 and M = 4.76, SD = 2.97, respectively), as well as the highest proportion of moderate-to-severe symptoms (≥4 in 67% and 65% of patients, respectively). This pattern highlights the predominance of physical symptom burden in this population, with fatigue and pain representing the most clinically relevant symptoms, consistent with the palliative radiotherapy setting. The ‘other symptoms’ category was highly heterogeneous, including gastrointestinal, vestibular, and neuropathic symptoms, with no single dominant symptom type identified. This item was completed by 14 patients, with reported severity ranging from 2 to 10 on the NRS scale. Mean values and distributions for each ESAS item are reported in Table 1.

### 3.1. Symptom Clusters Using PCA and K-means Clustering

The KMO measure of sampling adequacy indicated an acceptable level of appropriateness for factor analysis (overall MSA = 0.77), with individual item values ranging from 0.71 to 0.87. Bartlett’s test of sphericity was highly significant (*p* < 0.001), confirming that the correlation matrix was suitable for principal component analysis. Subsequently, PCA revealed four SCs, each accounting for over 10% of total variance. Taken together, the first four components explained a cumulative variance of 70%. In detail, SC_PCA_1 included tiredness, drowsiness, dyspnea, and malaise. SC_PCA_2 grouped depression and anxiety, SC_PCA_3 included nausea and loss of appetite, and SC_PCA_4 consisted solely of pain. K-means cluster analysis identified three clinically meaningful clusters: SC_KMC_1 (pain, tiredness, drowsiness and malaise), SC_KMC_2 (nausea, loss of appetite and dyspnea), and SC_KMC_3 (depression and anxiety) (see Tables S1–S3 and Figures S1–S3).

### 3.2. Associations Between SCs and Clinical Variables

Higher scores in SC_PCA_1, SC_PCA_3, and SC_PCA_4, as well as membership in SC_KMC_1, were significantly associated with worse ECOG PS (*p* < 0.05). Regarding the primary tumor site, a non-significant trend toward clustering was observed. Nevertheless, groups belonging to both SC_PCA_2 and SC_KMC_3 showed higher proportions of patients with breast and prostate cancer when compared with those not belonging to the clusters (*p* = 0.08 for both). Interestingly, liver metastases were significantly associated with SC_KMC_2, while a borderline association was observed with SC_PCA_3 (*p* = 0.06). Although not statistically significant, a trend toward association was noted between bone metastases and SC_PC_A4 (*p* = 0.07). Full results are presented in Table 2 and Table 3.

### 3.3. Associations with Palliative Radiotherapy

Among the 67 patients who received PRT, bone metastases were the most commonly treated sites (69%), followed by CNS (19%) and visceral metastases (12%). Single-fraction schedules were slightly more frequent than multi-fraction regimens (58% vs. 42%), with 8 Gy in a single fraction being the most commonly prescribed schedule. Additional details on radiotherapy regimens are reported in Table S4. In multivariable logistic regression, SC_PCA_2 was significantly associated with a lower likelihood of undergoing PRT (OR = 0.26, *p* < 0.05). The combination of SC_PCA_1 and 2 was associated with an increased probability of PRT administration (OR = 3.83, *p* = 0.06). Interestingly, gastro-enteric primary tumors were associated with an increase in PRT use (OR = 3.92, *p* < 0.05). Belonging to SC_KMC_3 was associated with a lower likelihood of receiving PRT (OR = 0.19, *p* < 0.05). When combined, SC_KMC_ 1 and 3 showed a non-significant trend toward higher PRT utilization (OR = 4.75, *p* = 0.07). ECOG PS 0-1 and locally advanced cancers were associated with higher likelihood of receiving PRT (OR = 5.38 and 3.99, respectively, *p* < 0.05 for both). Full model estimates are reported in Table 4.

### 3.4. Exploratory Survival Analysis

**Figure 1:**
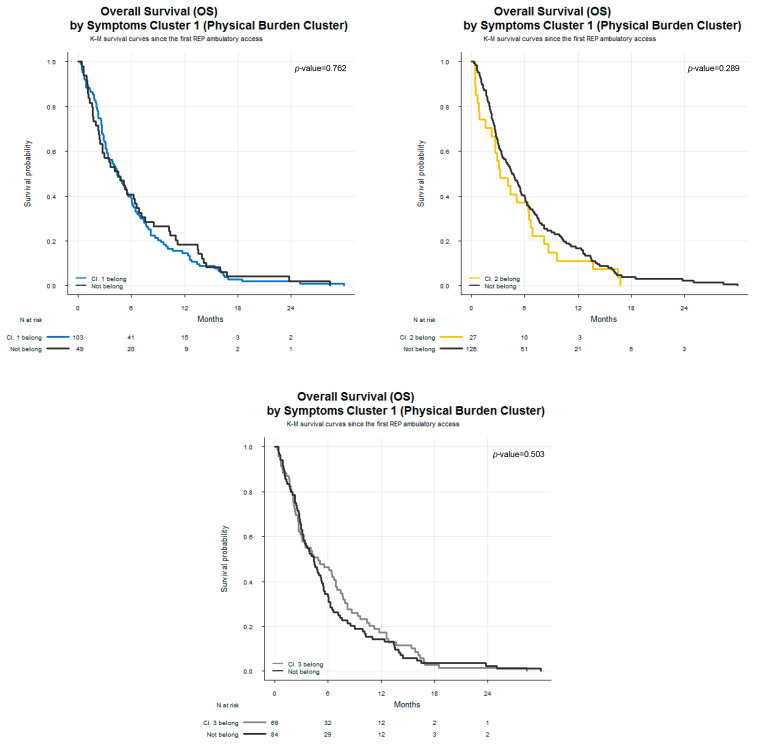
Survival analysis since the first REP ambulatory access by KMC symptoms cluster.

Even if not statistically significant, SC_KMC_2 was associated with a median OS of 3.22 months, compared to 4.63 months in those not belonging to the cluster (see Figure 1).

Furthermore, this trend toward shorter survival was observed in the SC_KMC_2 group at both 25th and 75th percentiles (see Tables S5 and S6 and Figure S4). Patients belonging to and not belonging to SC_PCA_1 had similar median OS values of 4.42 and 4.45 months, respectively. After accounting for clusters, age had an impact on OS with both PCA and KMC (*p* < 0.05 for both) (see Table S7). Notably, all patients considered for OS died during the observation period; therefore, no observations were censored in the survival analysis.

## 4. Discussion

PRT represents a cornerstone in the symptomatic management of patients with advanced-stage cancer. Nevertheless, estimating the clinical benefit of PRT—balancing symptom relief against potential treatment-related toxicity—remains challenging. In this context, a thorough evaluation of the patient’s physical condition, psychological status, and prognosis is essential for personalized treatment decisions. Over the last decade, several studies have highlighted the utility of a multidisciplinary and multidimensional approach, particularly emphasizing the collaboration between radiation oncologists and palliative care specialists in defining treatment indications and timing [11,24,25,26]. In Italy, the RaP outpatient clinic represents one of the few structured initiatives incorporating this collaborative model into routine clinical care [14].

Within this setting, we analyzed SCs with the aim of improving symptom monitoring and exploring their potential role in guiding therapeutic decisions. The PCA identified four clusters. SCPCA1, comprising tiredness, drowsiness, dyspnea, and malaise, corresponds to a systemic cluster. SCPCA2, including depression and anxiety, represents a psychological cluster. SCPCA3, composed of nausea and loss of appetite, reflects a gastrointestinal cluster. Finally, SCPCA4, defined solely by pain, suggests a distinct somatic experience not strongly correlated with other symptoms. The KMC analysis identified three clinically relevant SCs: SCKMC1 (pain, tiredness, drowsiness and malaise), representing a physical burden cluster; SCKMC2 (nausea, loss of appetite and dyspnea), representing a visceral discomfort cluster; and SCKMC3 (depression and anxiety), representing a psychological cluster. These findings reinforce the concept that patients with advanced cancer frequently present with interrelated symptoms rather than isolated complaints. Interestingly, pain emerged as a single-factor component in the PCA, whereas it clustered with dyspnea and malaise in the k-means ‘physical burden’ set. This may reflect the multifactorial nature and central clinical relevance of pain in advanced cancer, where it often represents a primary and independent driver of symptom burden potentially arising from both physical and psychological dimensions [27,28]. From a methodological perspective, this behavior may be explained by the fact that PCA captures dominant sources of variance, highlighting pain as a highly salient symptom, while k-means clustering identifies patterns of co-occurrence at the patient level. Consequently, it is not surprising that pain appears isolated in a dimensional reduction analysis like PCA, yet integrates into a broader SC in a pattern-based approach.

A consistent and clinically meaningful association emerged between ECOG PS and SCs characterized by physical symptoms. Specifically, both SCPCA1 and SCKMC1, characterized by fatigue-related and somatic symptoms, were significantly associated with worse ECOG PS. These results suggest that systemic symptom burden, especially symptoms such as tiredness, drowsiness, and malaise, is closely linked to impaired functional status in this patient population. Conversely, no significant association was observed between ECOG PS and psychological SCs (SCPCA2 and SCKMC3). This indicates that, in our cohort, psychological distress may compromise emotional well-being but does not substantially influence patients’ performance status. Our results are consistent with previous findings by Nieder et al. [29], who reported that nausea, fatigue, dry mouth, and appetite loss were significantly more prevalent in patients with poor PS. Although temporary symptom worsening post-PRT may occur in patients with poor PS, selected individuals—particularly those with dyspnea or pain—may still benefit from PRT. Similar results were also reported in older studies [30,31].

Although not statistically significant, we observed a trend—across both PCA and KMC models—suggesting an exploratory association between breast cancer and psychological SCs (SCPCA2 and SCKMC3, *p* = 0.08 for both). While this observation warrants cautious interpretation, it may hold clinical relevance. Hormonal dysregulation, common in breast cancer due to both disease and endocrine therapy, could increase susceptibility to psychological symptoms such as anxiety, depression, and fatigue [32,33]. Further research is needed to clarify this potential relationship.

We also identified meaningful symptom patterns according to metastatic sites. Liver metastases were significantly associated with the visceral discomfort cluster (SCKMC2), comprising nausea, loss of appetite, and dyspnea, with a borderline association for the gastrointestinal cluster (SCPCA3) (*p* = 0.06). These findings are consistent with previous evidence that hepatic involvement contributes to cachexia, metabolic dysfunction, anorexia, and fatigue, even before overt liver failure occurs [34,35,36]. A plausible explanation is the systemic inflammation and metabolic dysregulation induced by liver metastases, which may promote the co-occurrence of gastrointestinal and constitutional symptoms, in line with the pathophysiology of cancer-associated cachexia as a systemic inflammatory syndrome [37]. Conversely, a trend between bone metastases and the pain-specific cluster (SCPCA4), though not statistically significant (*p* = 0.07), remains clinically intuitive, reflecting the well-established pathophysiology of skeletal metastases—osteolysis, nerve compression, and inflammatory mediator release [38]. These trends may indicate a possible relationship between SCs, tumor biology, and metastatic burden. Larger prospective studies are warranted to validate these associations.

Our findings also highlighted a potential association between SCs and treatment decisions. In multivariable analysis, patients belonging to psychological clusters (i.e., SCPCA2 and SCKMC3) showed a significantly lower likelihood of receiving PRT. These consistent findings across two clustering methods suggest that psychological distress may contribute to lower referral rates, potentially reflecting physician perceptions of vulnerability and/or patient-related hesitancy due to emotional burden, low motivation, or treatment and travel-related fatigue. However, these interpretations should be considered with caution, as the study design does not allow differentiation between physician-driven decisions and patient refusal or preference regarding palliative radiotherapy. In this context, early referral to psycho-oncology could help address emotional distress, enhance coping, and support more balanced decision-making regarding palliative radiotherapy [39]. Conversely, when psychological clusters were combined with pain or a physical burden cluster (SCPCA4 and SCKMC1, respectively), they were associated with a higher likelihood of receiving PRT. However, these findings were not statistically significant at conventional thresholds and should therefore be interpreted with caution. They may suggest a possible interaction between psychological distress and physical symptom burden in influencing treatment decisions, but require confirmation in larger prospective studies. As expected, better ECOG PS were associated with a higher PRT use in our cohort. This is likely because patients with good PS are more suitable candidates for PRT, being able to better tolerate potential toxicities; moreover, ECOG PS has been shown to be the strongest predictor of survival after palliative RT [40]. Finally, the presence of locally advanced cancer emerged as an independent factor associated with a higher likelihood of receiving PRT. This is likely related to the important role that local treatments such as RT can play in this clinical context, regardless of the primary tumor site [41,42,43].

When comparing our results with previous studies exploring SCs in patients referred for PRT using ESAS scores, a number of consistent patterns emerge despite methodological differences. Several authors, including Chen et al. [44], Ganesh et al. [21] and McKenzie et al. [45], have applied various statistical techniques (PCA, EFA, HCA), consistently identifying symptom pairs such as anxiety and depression, nausea and loss of appetite, and tiredness and drowsiness as strongly interrelated. These recurring associations align closely with our findings, suggesting that certain symptom constellations may reflect stable and reproducible clinical phenomena rather than statistical artifacts.

Interestingly, while certain symptoms—such as nausea and loss of appetite or anxiety and depression—cluster together consistently across studies, others like pain, dyspnea, and malaise demonstrate greater variability depending on the population studied or the analytical method employed. Consequently, it is essential to emphasize that the derivation of SC from PCA should be guided by clinical reasoning, taking into account the pathophysiological and experiential interrelationships among symptoms. For symptom cluster analysis to be clinically meaningful, it must not only reflect consistent statistical associations but also provide practical utility in shaping care pathways and improving patient outcomes. Given that ESAS is routinely administered in PC settings, particularly by nursing staff, the identification of SCs may have relevant implications for nursing practice. Cluster-based interpretation of ESAS scores could support nurses in recognizing co-occurring symptom patterns, facilitating earlier identification of clinical deterioration and more structured prioritization of symptom management. Moreover, the use of SCs may enhance individualized care planning and improve communication within multidisciplinary teams involved in PRT pathways.

Our exploratory survival analysis suggested that patients in SCKMC2 (the visceral discomfort cluster) had a shorter median OS (3.2 vs. 4.6 months). Although this difference did not reach statistical significance due to the observational design and the limited size of the SCKMC2 subgroup, the trend may be clinically relevant. Notably, SCKMC2 comprises loss of appetite and dyspnea, which are the only two symptoms included in the validated PaP prognostic score for estimating short-term survival in advanced cancer patients [46,47]. The ProPaRT study further confirmed the prognostic value of these symptoms in patients selected for PRT [48]. Our results suggest that symptom clustering could serve as a potential proxy for latent prognostic trajectories; given the limited sample size and statistical power, this hypothesis warrants investigation in larger, prospective datasets.

This study has some limitations. Its cross-sectional design prevents assessment of symptom evolution over time or changes in SCs following PRT. Additionally, being a single-center study, the findings may not be generalizable to broader populations or different healthcare systems. While PCA and KMC are robust exploratory methods, cluster interpretation partially depends on clinical reasoning. Furthermore, some methodological choices were driven by the exploratory nature of the study. For instance, the choice of symptom cut-points (ESAS NRS ≥4 and the ‘≥ half items’ rule for cluster assignment), although consistent with previous symptom cluster studies and adopted to facilitate comparability across the literature, was applied post hoc and not derived from prospective validation; alternative thresholds or cluster assignment criteria may yield different classifications and associations. Similarly, multiple comparisons were performed across baseline characteristics without adjustment for multiplicity; therefore, statistically significant associations should be interpreted as exploratory and hypothesis-generating. Finally, although exploratory survival analysis was performed, the limited sample size and number of events precluded a statistically rigorous survival analysis, reducing the power to draw definitive prognostic conclusions. Future research should aim to validate these findings in independent and larger cohorts, ideally also using longitudinal designs to assess the stability of symptom clusters over time and their responsiveness to PRT and other supportive care interventions. Intuitively, such studies may further explain the role of symptom assessment in optimizing treatment planning in palliative care.

## 5. Conclusions

In conclusion, we identified SCs, by PCA and KMC, which are biologically plausible and consistent with previously reported symptom patterns. Physical symptoms-dominated clusters were associated with poorer ECOG PS and a higher likelihood of receiving PRT, whereas psychological clusters appeared linked to lower treatment rates. Although these findings should be interpreted with caution due to the retrospective design, they support the hypothesis that SC analysis may enhance clinical assessment and contribute to more personalized care in PRT settings. Prospective validation is warranted.

## Figures and Tables

**Table 1:** Summary statistics of baseline characteristics and ESAS items.

Variable	n	%
Radiation-oncologist-assessed ECOG performance status		
0–1	102	47
≥2	113	53
Palliativist-assessed ECOG performance status		
0–1	102	47
≥2	113	53
Sex		
Female	93	47
Male	122	53
Primary tumor site		
Breast	43	21
Lung	68	31
Prostate	26	12
Gastro-enteric	21	10
Genito-urinary	24	11
Other malignancies	33	15
Bone metastases		
No	45	21
Yes	170	79
Liver metastases		
No	182	85
Yes	33	15
Lung metastases		
No	165	77
Yes	50	23
Central nervous system metastases		
No	177	82
Yes	38	18
Soft tissue metastases		
No	206	96
Yes	9	4
Locally advanced primary tumor		
No	190	88
Yes	25	12
Other metastases		
No	135	63
Yes	80	37
Palliative radiotherapy		
No	148	69
Yes	67	31
Median age	71	
ESAS item	Total (n = 215)	
Pain	215	
Mean ± SD	4.76 ± 2.97	
Range	0–10	
% of scores ≥ 4	65	
Tiredness		
Mean ± SD	4.91 ± 3.04	
Range	0–10	
% of scores ≥ 4	67	
Drowsiness		
Mean ± SD	2.53 ± 3.01	
Range	0–10	
% of scores ≥ 4	33	
Nausea		
Mean ± SD	0.98 ± 2.23	
Range	0–10	
% of scores ≥ 4	12	
Loss of appetite		
Mean ± SD	2.10 ± 2.98	
Range	0–10	
% of scores ≥ 4	31	
Dyspnea		
Mean ± SD	1.31 ± 2.40	
Range	0–10	
% of scores ≥ 4	15	
Depression		
Mean ± SD	2.50 ± 2.88	
Range	0–10	
% of scores ≥ 4	33	
Anxiety		
Mean ± SD	2.44 ± 2.72	
Range	0–10	
% of scores ≥ 4	35	
Malaise		
Mean ± SD	1.97 ± 2.84	
Range	0–10	
% of scores ≥ 4	24	
Other symptoms		
Mean ± SD	0.48 ± 1.90	
Range	0–10	
% of scores ≥ 4	6	
ESAS Total score		
Mean ± SD	24.03 ± 15.28	
Range	0–80	

ECOG PS = Eastern Cooperative Oncology Group performance status, ESAS= Edmonton Symptom Assessment System, n = number, SD: standard deviation. The reported ranges reflect observed scores in the study population.

**Table 2:** Association between PCA symptom clusters and clinical characteristics.

Variable	PC 1					PC 2					PC 3					PC 4					*p*
	Belong		Not Belong		*p*	Belong		Not Belong		*p*	Belong		Not Belong		*p*	Belong		Not Belong		*p*	
	No.	%	No.	%		No.	%	No.	%		No.	%	No.	%		No.	%	No.	%		
Palliativist-assessed ECOG performance																					
0–1	35	36	67	56	**0.004**	48	48	54	47	0.777	26	36	76	54	**0.013**	59	42	43	57	**0.047**	0.252
≥2	61	64	52	44		51	52	62	53		47	64	66	46		80	58	33	43		
Bone metastases																					
No	22	23	23	19	0.520	19	19	26	22	0.563	20	27	25	18	0.095	24	17	21	28	0.074	0.336
Yes	74	77	96	81		80	81	90	78		53	73	117	82		115	83	55	72		
Liver metastases																					
No	79	82	103	87	0.389	88	89	94	81	0.111	57	78	125	88	0.055	117	84	65	86	0.792	0.282
Yes	17	18	16	13		11	11	22	19		16	22	17	12		22	16	11	14		
Lung metastases																					
No	71	74	94	79	0.385	76	77	89	77	0.994	57	78	108	76	0.739	111	80	54	71	0.144	0.760
Yes	25	26	25	21		23	23	27	23		16	22	34	24		28	20	22	29		
Central nervous system metastases																					
No	77	80	100	84	0.465	83	84	94	81	0.591	59	81	118	83	0.679	119	86	58	76	0.088	0.582
Yes	19	20	19	16		16	16	22	19		14	19	24	17		20	14	18	24		
Soft tissue metastases																					
No	92	96	114	96	0.990	94	95	112	97	0.559	68	93	138	97	0.162	132	95	74	97	0.400	0.890
Yes	4	4	5	4		5	5	4	3		5	7	4	3		7	5	2	3		
Locally advanced primary tumor																					
No	80	83	110	92	**0.038**	92	93	98	84	0.054	62	85	128	90	0.259	124	89	66	87	0.605	0.166
Yes	16	17	9	8		7	7	18	16		11	15	14	10		15	11	10	13		
Other metastases																					
No	56	58	79	66	0.225	60	61	75	65	0.540	39	53	96	68	**0.042**	73	60	52	68	0.207	0.812
Yes	40	42	40	34		39	39	41	35		34	47	46	32		56	40	24	32		

PCA = principal component analysis, PC= principal component, No. = number, *p* = *p* value, Bold font indicates statistical significance at α = 0.05.

**Table 3:** Association between k-means symptom clusters and clinical characteristics.

Variable	Cluster 1					Cluster 2					Cluster 3					*p*
	Belong		Not Belong		*p*	Belong		Not Belong		*p*	Belong		Not Belong		*p*	
	No.	%	No.	%		No.	%	No.	%		No.	%	No.	%		
Palliativist-assessed ECOG performance																
0–1	50	38	52	62	**0.001**	9	32	93	50	0.082	48	48	54	47	0.777	0.165
≥2	81	62	32	38		19	68	94	40		51	52	62	53		
Bone metastases																
No	29	22	16	19	0.587	9	32	36	19	0.118	19	19	26	22	0.563	0.345
Yes	102	78	68	81		19	68	151	81		80	81	90	78		
Liver metastases																
No	108	82	74	88	0.262	20	71	162	87	**0.037**	88	89	94	81	0.111	0.074
Yes	23	18	10	12		8	29	25	13		11	11	22	19		
Lung metastases																
No	100	76	65	77	0.859	21	75	144	77	0.815	76	77	89	77	0.994	0.981
Yes	31	24	19	23		7	25	43	23		23	23	27	23		
Central nervous system metastases																
No	108	82	69	82	0.955	20	71	157	84	0.105	83	84	94	81	0.591	0.311
Yes	23	18	15	18		8	29	30	16		16	16	22	19		
Soft tissue metastases																
No	123	94	83	99	0.079	25	89	181	97	0.064	94	95	112	97	0.559	0.547
Yes	8	6	1	1		3	11	6	3		5	5	4	3		
Locally advanced primary tumor																
No	114	87	76	90	0.441	24	86	166	89	0.638	92	93	98	84	0.054	0.299
Yes	17	13	8	10		4	14	21	11		7	7	18	16		
Other metastases																
No	76	58	59	70	0.070	12	43	123	66	**0.019**	60	61	75	65	0.540	0.240
Yes	55	42	25	30		16	57	64	34		39	39	41	35		

No. = number, *p* = *p* value, Bold font indicates statistical significance at α = 0.05.

**Table 4:** Factors associated with radiotherapy treatment initiation.

***Firth-Corrected Logit Model for Identifying Principal Components Associated with Radiotherapy Treatment***					
**Parameter**	**Adjusted** **OR**	**95% CI**		**Profile Penalized** **LRT ** ***p*** **-Value**	**Type III ** ***p***
		**Lower Limit**	**Upper Limit**		
***SC_PCA_ 1***	1.395	0.585	3.281	0.4481	0.4481
***SC_PCA_ 2***	0.264	0.066	0.803	0.0173	0.0173
***Primary tumor site (reference breast)***					0.2321
*Lung*	1.672	0.680	4.312	0.2653	
*Prostate*	1.218	0.364	3.925	0.7424	
*Gastro-enteric*	3.919	1.252	12.975	0.0188	
*Genito-urinary*	1.129	0.336	3.650	0.8405	
*Other malignancies*	1.159	0.396	3.394	0.7860	
***Locally advanced primary tumor***	2.425	0.996	6.076	0.0511	0.0511
***SC _PCA _1: SC _PCA _2***	3.829	0.929	19.128	0.0637	0.0637
*Likelihood Ratio Test (overall model fit)*	*χ^2^(9) = 26.91 *; *p = 0.0014*				
*Global Wald Test (all covariates)*	*χ^2^(9) = 40.75 *; *p < 0.0001*				
***Firth-Corrected Logit model for identifying (K-means) clusters associated with radiotherapy treatment***					
**Parameter**	**Adjusted** **OR**	**95% CI**		**Profile Penalized** **LRT ** ***p*** **-value**	**Type III ** ***p***
		**Lower limit**	**Upper limit**		
***SC_KMC_ 1***	1.544	0.698	3.457	0.2837	0.2837
***SC_KMC_ 3***	0.188	0.020	0.852	0.0278	0.0278
***Primary tumor site (reference breast)***					0.2258
*Lung*	1.736	0.705	4.484	0.2326	
*Prostate*	1.184	0.357	3.772	0.7761	
*Gastro-enteric*	3.987	1.280	13.152	0.0168	
*Genito-urinary*	1.149	0.341	3.715	0.8175	
*Other malignancies*	1.283	0.439	3.761	0.6458	
***Locally advanced primary tumor***	2.619	1.086	6.503	0.0322	0.0322
***SC _KMC _1: SC _KMC _3***	4.754	0.879	49.665	0.0725	0.0725
*Likelihood Ratio Test (overall model fit)*	*χ^2^(9) = 26.41; p = 0.0017*				
*Global Wald Test (all covariates)*	*χ^2^(9) = 39.53; p < 0.0001*				

OR = odds ratio, CI = confidence interval, LRT = Likelihood Ratio Test, PCA = principal component analysis, KMC = k-means clustering.

## Data Availability

The raw data supporting the conclusions of this article will be made available by the authors on request.

## Supplementary Materials

The following supporting information can be downloaded at https://www.mdpi.com/article/10.3390/medicina62071216/s1. Table S1: Eigenvalues and proportions of variance for components from the PCA.; Table S2: Loading factors from the PCA; Table S3: Cluster separation metrics; Table S4: Summary statistics of OS since the first REP ambulatory access by PCA symptoms cluster; Table S5: Summary statistics of OS since the first REP ambulatory access by KMC symptoms cluster; Table S6: Firth-Corrected Cox Proportional Hazards Models for identifying factors associated with Overall Survival; Table S7: Firth-Corrected Cox Proportional Hazards Models for identifying factors associated with Overall Survival; Figure S1: Biplots among components 1 to 4 from the PCA; Figure S2: Optimal number of clusters determination: Elbow method. Silhouette method and Gap Statistic; Figure S3: Combined cluster visualization and PCA biplots; Figure S4: Survival analysis since the first REP ambulatory access by PCA symptoms cluster.

## Abbreviations

The following abbreviations are used in this manuscript:

PRT	Palliative radiotherapy
SC	Symptom clusters
RaP	Radiotherapy and Palliative Care
ESAS	Edmonton Symptom Assessment System
PCA	Principal component analysis
KMC	K-means clustering
ECOG PS	Eastern Cooperative Oncology Group performance status
NRS	Numerical rating scale
AIC	Akaike Information Criterion
OR	Odds ratios
CI	Confidence interval
OS	Overall survival
No.	Number
n.	Number
SD	Standard deviation
p	P value
SE	Standard error
