# Supplementary material for: Symptom Clusters by Edmonton Symptom Assessment System in Radiotherapy and Palliative Care Clinic
Source: Medicina (Kaunas). 2026 Jun 23;62(7):1216. doi: 10.3390/medicina62071216 (PMC13413787; doi:10.3390/medicina62071216)
Supplement: Supplementary file 1 [file medicina-62-01216-s001.zip › medicina-4295845-supplementary.pdf]

Table S1. *Eigenvalues and proportions of variance for components from the PCA.*

PCA	Eigenvalue	Proportion	Cumulative
1	3.201748	0.3557	0.3557
2	1.128046	0.1253	0.4811
3	0.977049	0.1086	0.5896
4	0.949627	0.1055	0.6952
5	0.794355	0.0883	0.7834
6	0.648179	0.0720	0.8554
7	0.483856	0.0538	0.9092
8	0.452139	0.0502	0.9594
9	0.365001	0.0406	1.0000

Table S2.1. *Loading factors from the PCA (varimax rotation).*

Symptom	Component 1	Component 2	Component 3	Component 4
Pain	0.189301	0.205798	0.170293	**0.796603**
Tiredness	**0.762965**	0.224414	0.22392	0.083604
Nausea	0.147157	0.368723	**0.733906**	-0.08928
Depression	0.127723	**0.856198**	0.064731	0.174531
Anxiety	0.205678	**0.827394**	0.145361	0.010091
Drowsiness	**0.734788**	-0.04932	0.041778	0.349109
Loss of appetite	0.166618	-0.04658	**0.869242**	0.214205
Malaise	**0.660036**	0.289289	0.078702	-0.01671
Dyspnea	**0.538887**	0.107873	0.279936	-0.43031

Table S2.2. *Loading factors from the PCA (promax rotation).*

Symptom	Component 1	Component 2	Component 3	Component 4
Pain	0.175730	0.184298	0.181849	**0.800752**
Tiredness	**0.770536**	0.073065	0.058293	0.056134
Nausea	-0.077695	0.265581	**0.722201**	-0.075408
Depression	-0.032285	**0.923491**	-0.088856	0.188058
Anxiety	0.034735	**0.860438**	-0.023366	0.019620
Drowsiness	**0.848311**	-0.192106	-0.070715	0.315935
Loss of appetite	0.012499	-0.215437	**0.967813**	0.223678
Malaise	**0.668891**	0.185377	-0.102834	-0.041456
Dyspnea	**0.501380**	-0.034697	0.146159	-0.451972

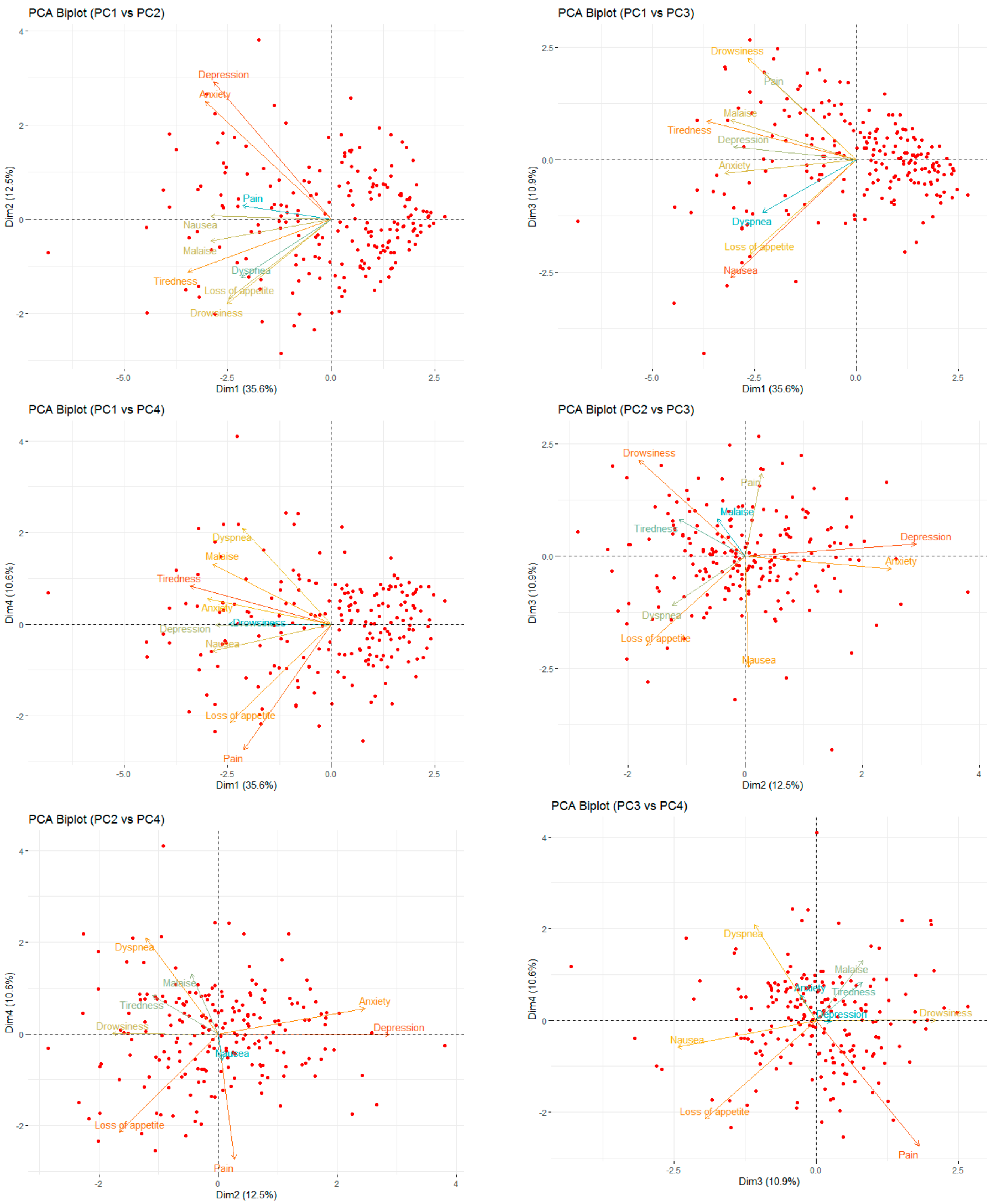

Figure S1. Biplots among components 1 to 4 from the PCA.

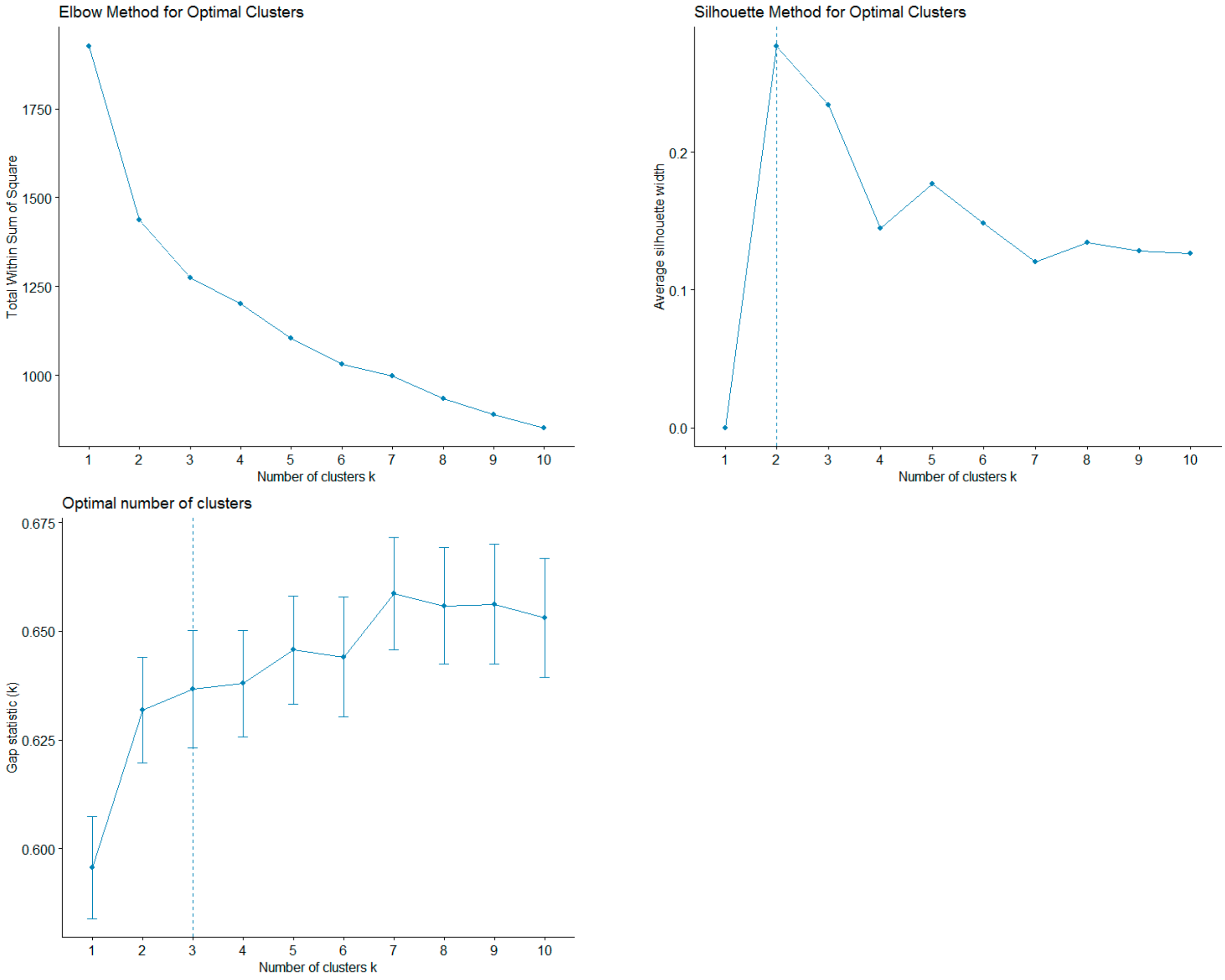

Figure S2. Optimal number of clusters determination: Elbow method. Silhouette method and Gap Statistic.

Table S3. *Cluster separation metrics.*

		R^2 Own Cluster	R^2 Next Cluster	Separation Metric
Cluster 1	Pain	0.146	0.146	1.000
	Malaise	0.267	0.259	0.990
	Tiredness	0.489	0.261	0.691
	Drowsiness	0.376	0.360	0.974
Cluster 2	Nausea	0.724	0.026	0.284
	Loss of appetite	0.310	0.209	0.871
	Dyspnea	0.149	0.044	0.890
Cluster 3	Depression	0.243	0.060	0.805
	Anxiety	0.342	0.250	0.877

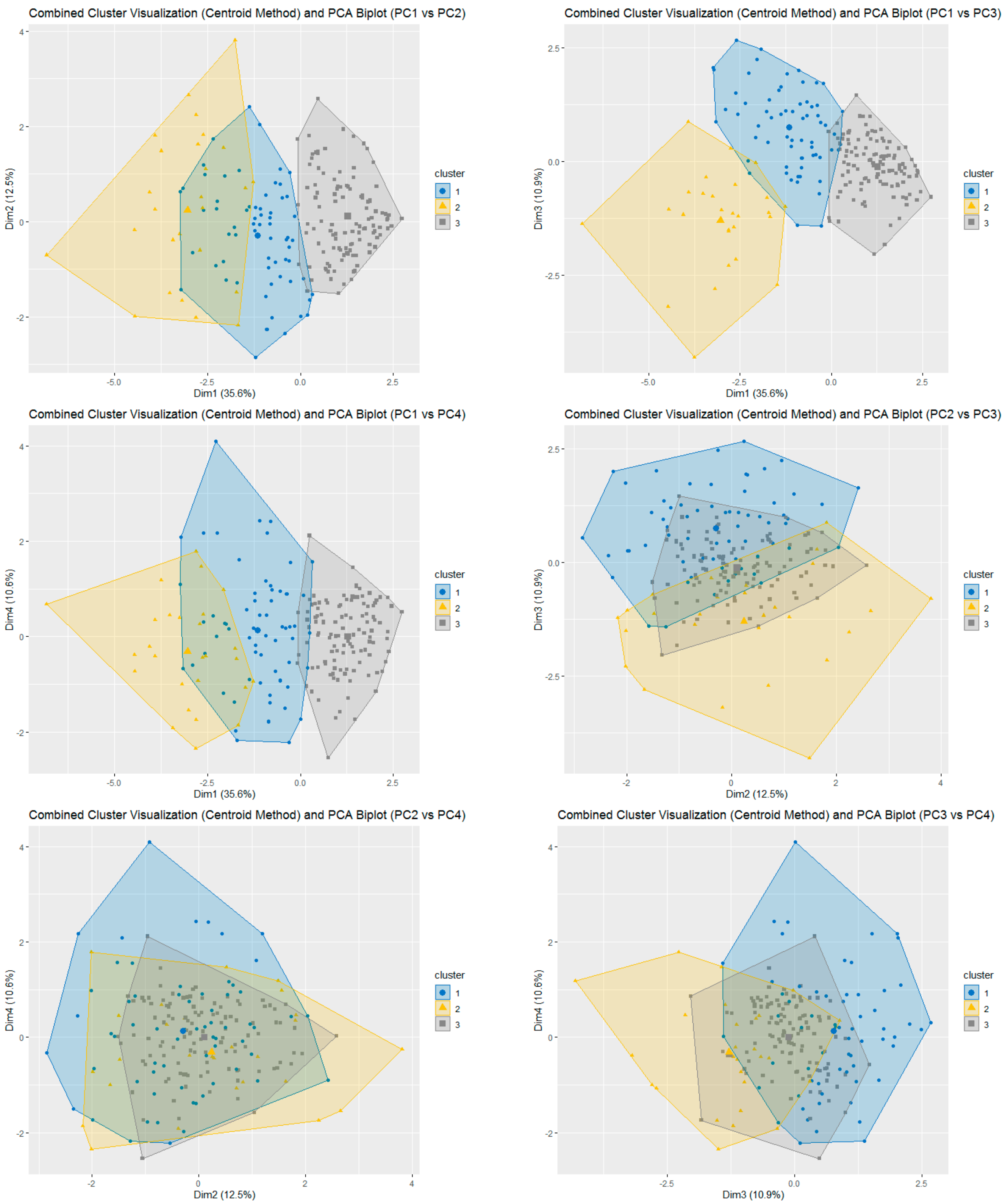

Figure S3. Combined cluster visualization and PCA biplots

Table S4. *Description of radiotherapy regimens.*

Palliative Radiotherapy details	No.	%
Treated patients	67	31
Treated anatomical sites		
Bone metastases	46	69
CNS	13	19
Visceral metastases	8	12
Fractionation schedule		
Multiple fractions	28	42
Single fraction	39	58
Irradiated doses		
400	19	28
500	5	7
600	2	3
700	3	4
800	38	57
Irradiated fractions		
1	39	58
3	4	6
5	24	36

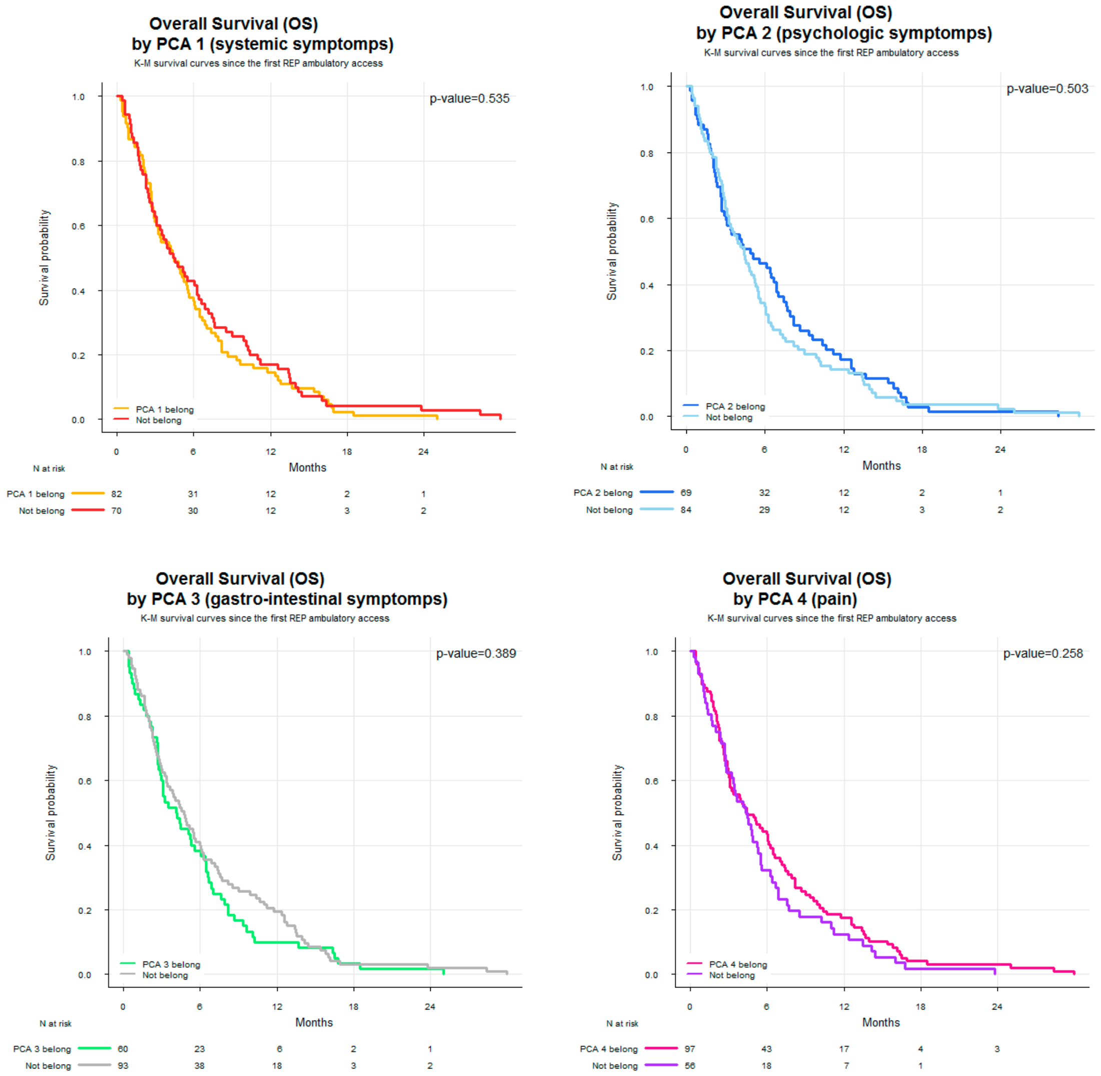

Figure S4. Survival analysis since the first REP ambulatory access by PCA symptoms cluster.

Table S5 . Summary statistics of OS since the first REP ambulatory access by PCA symptoms cluster.

	Median OS (95%CI) (*months*)	OS IQR (25p-75p)
PCA 1 belong	4.42 (3.22-5.65)	2.27 – 7.89
PCA 1 not belong	4.45 (3.12-6.57)	2.23 – 9.92
	Median OS (95%CI) (*months*)	OS IQR (25p-75p)
PCA 2 belong	4.86 (3.02-7.00)	2.14 – 9.36
PCA 2 not belong	4.39 (3.22-5.49)	2.33 – 7.24
	Median OS (95%CI) (*months*)	OS IQR (25p-75p)
PCA 3 belong	4.14 (2.96-6.41)	2.25 – 7.33
PCA 3 not belong	4.73 (3.38-6.08)	2.23 – 9.92
	Median OS (95%CI) (*months*)	OS IQR (25p-75p)
PCA 4 belong	4.40 (3.12-6.44)	2.23 – 9.00
PCA 4 not belong	4.37 (3.25-5.52)	2.14 – 6.87

Table S6. Summary statistics of OS since the first REP ambulatory access by KMC symptoms cluster.

	Median OS (95%CI) (*months*)	OS IQR (25p-75p)
KMC 1 belong	4.40 (3.35-6.01)	2.27 – 8.18
KMC 1 not belong	4.53 (4.53-2.76)	1.74 – 10.21
	Median OS (95%CI) (*months*)	OS IQR (25p-75p)
KMC 2 belong	3.22 (2.66-6.67)	0.89 – 6.87
KMC 2 not belong	4.63 (3.41-6.01)	2.27 – 8.51
	Median OS (95%CI) (*months*)	OS IQR (25p-75p)
KMC 3 belong	4.86 (3.02-7.00)	2.14 – 9.36
KMC 3 not belong	4.39 (3.22-5.49)	2.33 – 7.24

Table S7 . Firth-Corrected Cox Proportional Hazards Models for identifying factors associated with Overall Survival.

Firth-Corrected Cox Proportional Hazards Model for identifying PCA clusters associated with Overall Survival				
**Parameter**	**Hazard ratio (HR)**	**95% CI**		**Profile Penalized p-value**
		**Lower limit**	**Upper limit**	
SC_{PCA} 1	1.070	0.734	1.564	0.7253
SC_{PCA} 2	0.829	0.580	1.187	0.3063
SC_{PCA} 3	1.105	0.771	1.576	0.5849
SC_{PCA} 4	1.253	0.882	1.765	0.2051
Age (1 year increase)	1.016	1.001	1.031	0.0375
ECOG-PS (0-1 vs. 2-4)	0.776	0.530	1.126	0.1832

Likelihood Ratio Test (overall model fit) *$\chi^2(6) = 12.30; p = 0.0556$*
Global Wald Test (all covariates) *$\chi^2(6) = 11.98; p = 0.0623$*

Firth-Corrected Cox Proportional Hazards Model for identifying k-means clusters associated with Overall Survival				
**Parameter**	**Hazard ratio (HR)**	**95% CI**		**Profile Penalized p-value**
		**Lower limit**	**Upper limit**	
SC_{KMC} 1	1.038	0.730	1.493	0.8386
SC_{KMC} 2	1.196	0.765	1.808	0.4210
SC_{KMC} 3	0.806	0.575	1.130	0.2107
Age (1 year increase)	1.017	1.002	1.033	0.0298
ECOG-PS (0-1 vs. 2-4)	0.802	0.546	1.164	0.2473

Likelihood Ratio Test (overall model fit) *$\chi^2(5) = 11.02; p = 0.0509$*
Global Wald Test (all covariates) *$\chi^2(5) = 10.60; p = 0.0560$*
